# COX5A Plays a Vital Role in Memory Impairment Associated With Brain Aging *via* the BDNF/ERK1/2 Signaling Pathway

**DOI:** 10.3389/fnagi.2020.00215

**Published:** 2020-07-10

**Authors:** Yan-Bin Xiyang, Ruan Liu, Xu-Yang Wang, Shan Li, Ya Zhao, Bing-Tuan Lu, Zhi-Cheng Xiao, Lian-Feng Zhang, Ting-Hua Wang, Jie Zhang

**Affiliations:** ^1^Institute of Neuroscience, Basic Medical College, Kunming Medical University, Kunming, China; ^2^Department of Neurosurgery, Shanghai Jiao Tong University Affiliated 6th People’s Hospital, Shanghai, China; ^3^Monash Immunology and Stem Cell Laboratories (MISCL), Monash University, Clayton, VIC, Australia; ^4^Key Laboratory of Human Diseases Comparative Medicine, Ministry of Health, Institute of Laboratory Animal Science, Chinese Academy of Medical Sciences (CAMS) and Comparative Medicine Centre, Peking Union Medical College (PUMC), Beijing, China; ^5^Yunnan Provincial Key Laboratory for Birth Defects and Genetic Diseases, Department of Medical Genetics, The First People’s Hospital of Yunnan Province, Affiliated Hospital of Kunming University of Science and Technology, Kunming, China

**Keywords:** COX5A, brain senescence, memory impairment, mitochondria, BDNF, ERK1/2

## Abstract

Cytochrome c oxidase subunit Va (COX5A) is involved in maintaining normal mitochondrial function. However, little is known on the role of COX5A in the development and progress of Alzheimer’s disease (Martinez-Losa et al., 2018). In this study, we established and characterized the genomic profiles of genes expressed in the hippocampus of Senescence-Accelerated Mouse-prone 8 (SAMP8) mice, and revealed differential expression of COX5A among 12-month-aged SAMP8 mice and 2-month-aged SAMP8 mice. Newly established transgenic mice with systemic COX5A overexpression (51% increase) resulted in the improvement of spatial recognition memory and hippocampal synaptic plasticity, recovery of hippocampal CA1 dendrites, and activation of the BDNF/ERK1/2 signaling pathway *in vivo*. Moreover, mice with both COX5A overexpression and BDNF knockdown showed a poor recovery in spatial recognition memory as well as a decrease in spine density and branching of dendrites in CA1, when compared to mice that only overexpressed COX5A. *In vitro* studies supported that COX5A affected neuronal growth *via* BDNF. In summary, this study was the first to show that COX5A in the hippocampus plays a vital role in aging-related cognitive deterioration *via* BDNF/ERK1/2 regulation, and suggested that COX5A may be a potential target for anti-senescence drugs.

## Introduction

Brain senescence, an age-related moderately advanced and irreversible condition, often leads to learning deficits and memory deterioration (Vanguilder and Freeman, 2011). In the advanced stage, it can manifest as Alzheimer’s disease (AD) and other age-related clinical conditions. Despite an intense effort and billions of dollars invested in research in the field of neuroscience, current therapeutic measures for the prevention and treatment of brain senescence are limited and far from satisfactory (Farzaei et al., 2019). Therefore, further investigation is required to reveal details of brain senescence and the associated impairment in cognitive functions.

With the deepening of research on brain senescence, memory impairment is considered one of the most prominent consequences of aging, and memory deterioration starts with the advancement of age (Zhao et al., 2011). Both clinical and experimental studies have shown that the hippocampus plays a very important role in learning, memory, and cognitive functions (Bussey et al., 2001; Daselaar et al., 2001).

The impaired mitochondrial dynamics of cytochrome c oxidase (COX) and/or mitochondrial malfunction are vital events involved in the progression of AD. Mitochondria are highly dynamic organelles, ranging from giant tubular networks to small round entities through rapid and reversible fission and fusion processes (Simoncini et al., 2015; Nitzan et al., 2019). In previous studies, it was demonstrated that mitochondrial dysfunction is associated with almost all neurodegenerative diseases and neurodegenerative-related events (Fivenson et al., 2017). Also, reports have revealed that impaired mitochondrial dynamics in favor of fission occurred in the hippocampal tissue of AD patients (Frazier et al., 2006; Detmer and Chan, 2007) Moreover, it has been demonstrated that AD is no exception, and data have suggested that mitochondrial malfunctioning included improper organelle dynamics, defective oxidative phosphorylation, oxidative stress, and harmful beta-amyloid associations (Hroudova et al., 2014; Dos Santos et al., 2019). In other studies, it was revealed that a major change that often associated with AD involved impairment of the mitochondrial electron transport chain at complex IV: cytochrome c oxidase (Omori et al., 2017). Thus, mitochondrial damage, such as COX I and IV inhibition, might play a role in facilitating tangle formation and, thereby, neurodegeneration (Melov et al., 2007). Furthermore, the nuclear-encoded subunits of COX, including subunit Va (COX5A), have been thought to be important in the regulation of age-related oxidative phosphorylation (Xiyang et al., 2016). In the present study, numerous differentially expressed COX genes were identified in senescence-accelerated mouse-prone 8 (SAMP8) and control genes from the hippocampal genomic maps. One of these genes was identified as COX5A. However, the possible roles of COX5A in the memory deficiency associated with brain senescence and age-related diseases, as well as its underlying signaling pathway are still not well understood.

To further explore the possible roles of COX5A in brain senescence, we established an up-regulated COX5A transgenic (Tg) mouse model. Alterations of spatial learning and memory and hippocampal synaptic plasticity [by electrophysiological long-term potentiation (LTP) and morphology detection; Fivenson et al., 2017] were determined *in vivo* and *in vitro*. Also, to determine whether COX5A exerted its effects *via* BDNF, cultured neurons from mouse hippocampi were treated with COX5A-ORF, and BDNF was knocked down. Double Tg mice, characterized by both COX5A up-regulation and BDNF knockdown, were used to confirm the BDNF-dependent role of COX5A. The findings in the present findings may provide novel evidence to explore the role of COX5A, its underlying BDNF-regulating mechanisms, as well as its roles in the aging brain.

## Materials and Methods

### Ethics Statement

Animal use and care were following the animal care guidelines, which conformed to the Guide for the Care and Use of Laboratory Animals published by the US National Institutes of Health (NIH Publication No. 85-23, revised 1996) and the Care and Use Guidelines of Experimental Animals established by the Ministry of Medicine of Yunnan, China. The ethics committee of the Kunming Medical University in Yunnan, China specifically approved this study (permit number: km-edw-2013118). All surgical procedures were performed using 5% isoflurane and were maintained at 1 to 2% isoflurane. All efforts were made to minimize suffering.

### Animal Grouping and Sample Preparation

Animal grouping and experimental procedures were performed as described in Supplementary Figure S1.

#### Sample Preparation

The senescence-accelerated mouse (SAM) is an aging model obtained by several generations of sister-brother breeding from litters of AKR/J mice (Takeda et al., 1981). SAMP8, an autogenic senile strain characterized by early cognitive impairment and age-related deterioration of learning and memory, has become a major biogerontological resource in aging research for which SAM-resistant 1 (SAMR1) serves as a control (Takeda et al., 1981; Nakahara et al., 1998).

Samples of the hippocampus were collected from five 2-month-old SAMP8 and five 12-month-old SAMP8 mice, and RNA was isolated using TRI reagent (Gibco Life Technologies, Rockville, MD, USA). Total RNA from each sample was quantified by fluorescer and Horest33258 dye, and RNA integrity was assessed by standard denaturing agarose gel electrophoresis. The total RNA of each sample was used for labeling and array hybridization.

#### RNA Labeling and Array Hybridization

The Mouse 12x135K Gene Expression Array was manufactured by Roche NimbleGen (Roche NimbleGen, Madison, WI, USA). Double-stranded cDNA (ds-cDNA) was synthesized from total RNA using an Invitrogen SuperScript ds-cDNA synthesis kit (Invitrogen, Carlsbad, CA, USA) in the presence of 100 pmol oligo dT primers. The ds-cDNA was cleaned and labeled following the NimbleGen Gene Expression Analysis protocol (NimbleGen Systems, Inc., Madison, WI, USA).

### Western Blot Analysis

To investigate the levels of multiple proteins, either the hippocampus of mice with different genotypes or cultured cells were prepared as described before Xiyang et al. (2016). Briefly, proteins were transferred from the gel to a nitrocellulose membrane (Thermo Fisher Scientific, Waltham, MA, USA). Subsequently, membranes were incubated with antibodies directed against COX5A (1:1000, Chemicon), BDNF (1:200, Chemicon), extracellular signal-regulated kinase 1/2 (ERK1/2, 1:400, Santa Cruz Biotechnology, Santa Cruz, CA, USA), and phosphorylated ERK1/2 (1:400, Santa Cruz Biotechnology, Santa Cruz, CA, USA), at 4°C for 24 h. After, membranes were washed four times with Tris Buffered Saline Tween (TBST) for 10 min each. Then, membranes were incubated with horseradish peroxidase (HRP)-conjugated secondary antibody (1:1,000; Vector Laboratories, Burlingame, CA, USA) for 2 h at room temperature. After washed three times with TBST for 10 min each, membranes were developed using an ECL kit, then imaged using a Bio-Gel Imaging system equipped with Genius synaptic gene tool software. Densitometry analyses for target proteins were performed. GAPDH (1:800, Santa Cruz Biotechnology, Santa Cruz, CA, USA) was used as an internal control.

### Immunofluorescence Analysis

To evaluate the effects of COX5A on the nervous system, immunolabeling with specific markers (NeuN, GFAP, and NF) was implemented. Also, hippocampal COX5A colocalization with neurons, and BDNF was performed. Antifade mounting Medium (Beyotime, Shanghai, China) containing DAPI was used to counterstain the nuclei. Images were acquired using a fluorescent microscope (LEICA DMI6000B, Germany). Details are described in the Supplementary Material.

### RT-PCR

RT-PCR was performed according to the protocol described by Xiyang et al. (2016). Briefly, an equal amount of RNA (4 μg) was used for each experiment, and multiple genes were determined by RT-PCR. GAPDH was used as a reference and subtracted for net changes. Gene primers were synthesized by the TaKaRa Company (TaKaRa, Osaka, Japan). RT-PCR products were electrophoresed and visualized using an ultraviolet gel imager (BIO-GEL, BIO-RAD, Hercules, CA, USA). The gene names and primer sequences are summarized in Table 1. Image analysis was performed using the SYN Gene Tool (Life Sciences, USA). The grayscale of each objective band was determined and estimated by comparing the relative intensity to that of GAPDH.

**Table 1:** Primer names, sequences and PCR conditions used for RT-PCR.

**Genes Primer sequence TmoC Product**
BDNF forward: 5′ ATG GGA AAT GAG GCG AGT T 3′ 52 479bp
reversal: 5′ AGG CCA CCT ACG ACA GCA C 3′
ERK1/2 forward: 5′ AGC GGC ACT CCA TCA TCT CGG 3′ 53 622bp
reversal: 5′ AGG AAG GGC TTG AGC GGG TAG G 3′
COX5A forward: 5′ GGT CAG AAC CCA ACA GAA G 3′ 52 287bp
reversal: 5′ TGT CCG TCG CCA TCA ATA T 3′
GAPDH forward: 5′ GTC CTT GAT CAC CCG ATT C 3′ 58 489bp
reversal: 5′ TCC TGT GTG CTT TCC ATT C 3′

### Cell Models

#### Vector Construct

Primers, plasmid vectors, and restriction enzymes used for vector construction were produced by GeneCopoeia™ (USA). First, COX5A cDNA was connected to the pReceiver-Lv130 plasmid vector fused to the Enhanced Green Fluorescent Protein (EGFP). Newly developed cDNA was inserted between an NspV restriction site 5′ to the COX5A gene (5′-GCTTGGAAGGAGTTCGAACCATG-3′) and an XhoI restriction site 3′ to the gene (5′-ACGCCGGCGTGAGCTCCAT-3′).

Full-length open reading frame (ORF) cDNA clones for COX5A gene [purchased from Origene (Rockville, MD, USA)] were directionally cloned into the pReceiver-Lv130 plasmid vector. COX5A sequences were obtained by RT-PCR as described above. The full length of ORF is 2849bp. Then, the cytomegalovirus (CMV) immediate early gene promoter was inserted downstream in the vector, using a newly created SpeI/XmnI restriction site. The product was determined by the TaKaRa Company (Osaka, Japan) production number: CCS-Q0450-Lv130 ORF.

Four potential shRNA sequences were designed for targeting the COX5A mRNA (NCBI Accession Number: GeneID: 12858) to silence the expression of COX5A. Each designed shRNA sequence included a segment of an shRNA sequence between the sense strand (5′-CCGACAACCACTACCTGA-3′) and anti-sense strand (5′-CGTGAAGA- ATGTGCGAGAC-3′) and was separated by a Hairpin Loop (5′-TCAAGAG-3′). The resulting DNA fragments were inserted between the BamHI and EcoRI restriction sites and fused to a psiHIV-U6 (HIV based) plasmid vector. The transfection of PC12 cells was used for shRNA screening.

#### Lentiviral Vector Production and Transduction

Lentiviral vector particles were produced by transfection of 293 Tα cells. 293 Tα cells were cultured with adequate nutrition. Once P4 cells were 60–70% confluent, 293 Tα cells were transfected using a vector plasmid encoding COX5A or silencing COX5A and a packaging plasmid composed of group antigen gene (gag), polymerase gene (pol) and envelope protein (env).

Cultured neurons obtained from neonatal C57BL/6J mice were prepared and treated as described as follows: blank (normal), phosphate buffer saline (PB), vector-ORF (control-ORF), COX5A-ORF, vector-shRNA (control-shRNA), and COX5A-shRNA. Neurons transfected with blank, PB, Vector-ORF, and Vector-shRNA served as controls. Briefly, 6 days after seeding, neurons were transduced with the packaged lentivirus (10 μl) and polybrene (3 μl) in DMEM. Cells were incubated for 2 h at 4°C, then for 12 h at 37°C and 5% CO_2_. The medium was removed and replaced with the N + B27 medium. Images at 200× magnification were acquired 2 days after transduction. LEICA DMI6000B (LAS AF system) was used for measuring the length of axons, areas of neurons, and cell number.

### Animal Models

COX5A-UP, BDNF-KD, and COX5A-UP/BDNF-KD Tg mice on a C57BL/6J genetic background were established by our collaborators in The Institute of Laboratory Animal Science (Chinese Academy of Medical Sciences and Comparative Medicine Centre, Peking Union Medical College, Beijing, China) and described in the Supplementary Materials. In this study, Tg mice and their age-matched, non-Tg littermates (wild-type, WT mice) were used. Mice were housed with free access to food and water in an environment with a 12-h light/dark cycle.

### Mitochondria COX Activity Assay and ATP Measurement

The preparation of mitochondria was described following the manufacturer’s guidelines (Sigma–Adrich, St. Louis, MO, USA). The protein concentration was determined using the BCA protein assay (Thermo Laboratories). Prepared 60 μg mitochondrial fractions were processed for COX activity assay detection. The COX activity of mitochondrial fractions was measured using the Cytochrome c Oxidase kit (Sigma–Adrich, St. Louis, MO, USA).

The ATP Colorimetric/Luminescence Assay kit (BioVision Incorporated, Milpitas, South Milpitas Blvd. Milpitas, CA, USA) was used to quantify hippocampal mitochondrial ATP content.

### Morris Water Maze Test

The Morris water maze (MWM) consisted of a circular pool (100 cm diameter, 50 cm deep) filled with water at 24–26°C to a depth of 20 cm. The MWM test was performed following the procedure described by Xiyang et al. (2016).

### Electrophysiology

To determine whether hippocampal synaptic plasticity was affected by COX5A up-regulation or BDNF knockdown, systematic analysis of synaptic functions was performed in the hippocampus as described by Xiyang et al. (2016). Briefly, all mice went through a preliminary trial, a day before the regular testing. This was to acclimatize them to water and expose them to the presence of a platform for an escape from the water. For this trial, a mouse was placed in the water for 10 s, allowed to swim around, and then placed on a platform submerged underwater for only 1–2 s. For regular trials, the hidden platform was put in a different location. Trials were conducted for five consecutive days, three times each morning and afternoon, and the escape latencies were recorded. On the afternoon of the 5th day, the platform was removed for the probe trial and all mice were allowed to swim for 60 s to assess their memory for the platform location. The time spent and distances traveled in the four quadrants were noted.

### Golgi Staining

The brains of COX5A-UP Tg mice, 6-months-old and 18-months-old, were rapidly removed. FD Rapid Golgi stain kits (FD Neuro Technologies, Baltimore, MD, USA) were used for Golgi staining. Neurons were chosen based on criteria that were described previously (Hoffman et al., 2011). The diameter of somata, basal/apical branch points and the average dendritic spine number (spine density, per 100 μm) were also quantified.

### Changes in BDNF Expression and Associated Signaling After COX5A Up-Regulation

Mice were anesthetized by isoflurane as described before. The brains were rapidly removed and placed into ice-cold phosphate-buffered saline (PBS) for hippocampus extraction. Hippocampi were harvested for RT-PCR. Then, RT-PCR for BDNF and ERK1/2 genes was performed according to the procedure described before (refer to “RT-PCR” in “Materials and Methods” section in this study). Primers used are listed in Table 1. Primers were synthesized by TaKaRa Company (Osaka, Japan). Western blot analysis was employed to analyze protein levels of BDNF, ERK-1, and p-ERK1/2.

### BDNF Rescue Setting

BDNF cDNA was ligated into the Receiver-Lv127 plasmid vector fused to the EGFP. BDNF sequences were obtained by RT-PCR, using the following primers: sense, 5′-ATCCACGCTGTTTTGACC-3′, anti-sense, 5′-CCGGACACGCTGAACTTGT-3′. The newly established cDNAs were inserted between an NspV restriction site 5′ to the BDNF gene (5′-GCTTGGAAGGAGTTCGAACCATG-3′) and an XhoI restriction site 3′ to the gene (5′-ACGCCGGCGTGAGCTCCAT-3′).

To silence the expression of BDNF, four potential shRNA sequences targeting BDNF mRNA (NCBI accession number: GeneID: 627) were designed. Each designed shRNA sequence included a segment of the shRNA sequence between the sense strand (5′-CCGACAACCACTACCTGA-3′) and the anti-sense strand (5′-CGTGAAGA-ATGTGCGAGAC-3′), separated by a hairpin loop sequence (5′-TCAAGAG-3′). The specific piece of DNA was synthesized by GeneCopoeia^TM^ (Maryland, MD, USA), and the resulting DNA fragments were inserted between the BamHI and EcoRI cloning sites and cloned into the psiHIV-U6 plasmid vector.

Lentiviral vector particles were produced by transfection of 293 Tα cells. Once cells of passage p4 were 60–70% confluent, 293 Tα cells were transfected with a vector plasmid encoding BDNF or silencing BDNF and a packaging plasmid composed of gag, pol, and env.

Cultured neurons were grouped into: normal (blank), PB, Vector-ORF (Control-ORF), COX5A-ORF, BDNF-ORF, Vector-shRNA (Control-shRNA), COX5A-shRNA, BDNF-shRNA. Neurons treated with blank, PB, Vector-ORF, and Vector-shRNA served as controls (Supplementary Material). Additionally, COX5A-ORF or COX5A-shRNA+ BDNF-ORF transfected neurons were pretreated with a specific ERK1/2 inhibitor, PD98059 (Sigma–Adrich, St. Louis, MO, USA) at 5 μM for 30 min. To evaluate whether COX5A could exert its effects *via* BDNF/ERK1/2 signaling, neurite length, number, and areas of cultured cells were determined.

### Statistical Analyses

For gene array, slides were scanned at 5 μm/pixel resolution using an Axon GenePix 4000B scanner (Molecular Devices Corporation, San Jose, CA, USA) piloted by GenePix Pro 6.0 software. Scanned images were imported into NimbleScan software (version 2.5) for grid alignment and expression data analysis. Expression data were normalized through quantile normalization and the Robust Multichip Average (Regan et al., 2019) algorithm in the NimbleScan software. All gene-level files were imported into Agilent GeneSpring GX software (version 11.5.1, USA) for further analysis. Differentially expressed genes were identified through fold change filtering. Hierarchical clustering was performed using Agilent GeneSpring GX software. GO analysis and pathway analysis was performed using the standard enrichment computation method.

The software package SPSS version 19.0 for Windows covariance was employed for statistical analysis of other data. The results are expressed as the mean ± standard deviation (SD). Differences between the two groups were evaluated using Student’s *t*-test. When analyzing one-variable experiments with >2 groups, the significance of the difference was evaluated using ANOVA followed by Bonferroni’s *post hoc* tests. Two-way repeated-measures (RM) ANOVA followed by Tukey’s test were employed for the MWM test and electrophysiology analysis. *P ≤ 0.05* was considered significant.

## Results

### Screening of Differentially Expressed Genes

**Figure 1:**
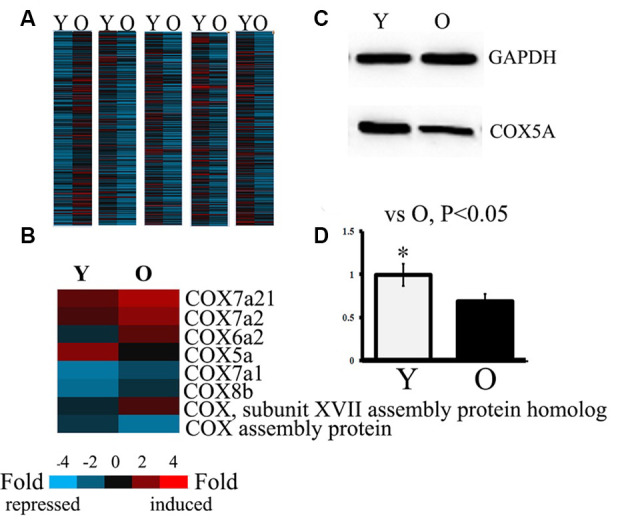
Analysis and identification of gene assays in both young and old Senescence-Accelerated Mouse-prone 8 (SAMP8) mice. Y, young SAMP8 (2-months-old); O, old SAMP8 (12-months-old). Panels **(A,B)** show the heat map analyzed by hierarchical clustering for all targets. “Red” indicates high relative expression, and “blue” indicates low relative expression. Panel **(A)** shows differentially expressed genes by fold change filtering between young and aged SAMP8 mice. The threshold fold change ≥ 2.0. Panel **(B)** shows the differentially expressed genes belonging to COX subunits and COX functional units in the hippocampus of young and aged SAMP8 mice. **(C)** Representative protein bands as detected by Western blot analysis (WB). Lane Y, protein expression in young SAMP8 mice; Lane O, protein expression in old SAMP8 mice. **(D)** The quantitative analysis of the COX5A expression.

To identify differentially expressed genes, Fold Change filtering was performed between samples. The threshold was set to Fold Change ≥ 2.0. A total of 12,389 differentially expressed genes (7,307 genes up-regulation, 5,182 genes down-regulation) were detected in genomic maps of hippocampi between the young and aged SAMP8 mice. Eight differential genes belonging to COX subunits and COX functioning were detected between hippocampi of young vs. aged SAMP8 mice (Figures 1A,B). These were designated COX subunit VIIa polypeptide 2-like (COX7A2l), COX subunit VIIa 2 (COX7A2), COX subunit VI a, polypeptide 2 (COX6A2), COX subunit Va (COX5A), COX subunit XVII (COX17) assembly protein homolog, COX subunit VIIa 1 (COX7A1), COX subunit VIIIb (COX8B), COX15 homolog, and COX assembly protein. Quantitative analysis revealed that the levels of COX7A2l, COX7A2, COX6A2, COX17 assembly protein homolog, COX7A1, and COX8B were significantly increased, while mRNA of COX5A and COX15 homolog were markedly decreased in aged SAMP8 mice. Considering that COX5A is important in the regulation of age-related oxidative phosphorylation (Arnold et al., 1998), this gene was chosen as the target. Western blot analysis validated that the protein level of COX5A was down-regulated in aged SAMP8 mice when compared with that of young mice (Figures 1B–D).

### COX5A Promotes Neurite Outgrowth in Hippocampal Neurons *in vitro*

**Figure 2:**
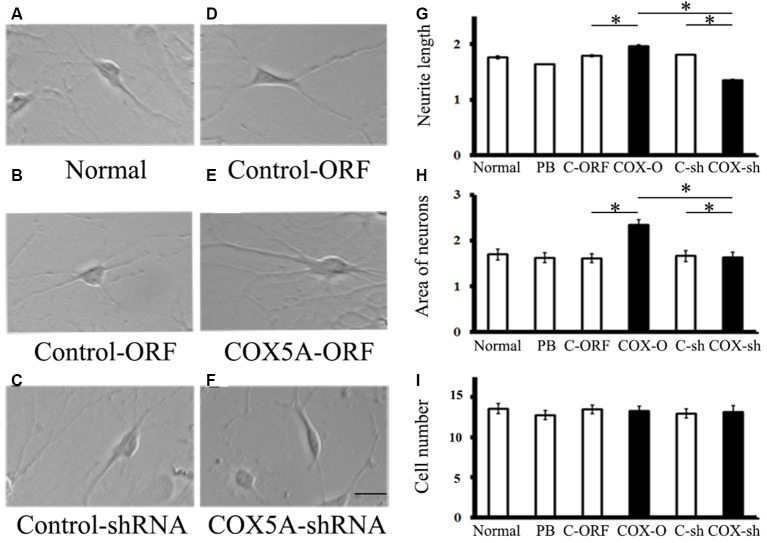
**(A,B)** Effects of COX5A on neurite outgrowth in hippocampal neurons *in vitro*. **(C–F)** Morphology and axonal length of hippocampal neurons transfected with different constructs. **(G–I)** Quantification of neurite extensions. Evaluations of neurite length **(G)** areas of cultured neurons **(H)** and the number of cells **(I)** after introduction with COX5A-ORF or COX5A-shRNA. Values plotted to represent the mean ± SD. *P < 0.05, by one-way analysis of variance. C-ORF, control ORF; COX-O, COX5A-ORFC-sh, COX-sh.

To investigate the role of COX5A in regulating neurite outgrowth, we designed an overexpression construct to up-regulate COX5A expression and an shRNA construct to knockdown COX5A expression in cultured hippocampal neurons. COX5A-ORF transduced into hippocampal cells induced a marked outgrowth in neurite extensions (*F* = 16.51, *P* = 0.0003, *P* < 0.05, *n* = 5) and neuron area (*F* = 18.33, *P* = 0.00012, *P* < 0.05, *n* = 5) when compared with the Control-ORF group (Figures 2C,D,G). Conversely, down-regulation of COX5A by transfection with COX5A-shRNA in cultured hippocampal neurons decreased both neurite extensions (*F* = 13.56, *P* = 0.000, *P* < 0.05, *n* = 5) and the neuron area (*F* = 20.13, *P* = 0.000, *P* < 0.05, *n* = 5) when compared with the control-shRNA-treated group (Figures 2E,F,H). However, neither administrating COX5A-ORF (*P* = 0.074, *P* > 0.05) nor COX5A-shRNA (*P* = 0.068, *P* > 0.05) affected the number of cells (Figure 2I). Furthermore, no significant differences were observed among normal, PB-treated cells, and control cells (Figures 2A–C,E, *P* < 0.05).

### Effects of Over-Expressing COX5A *in vivo*

**Figure 3:**
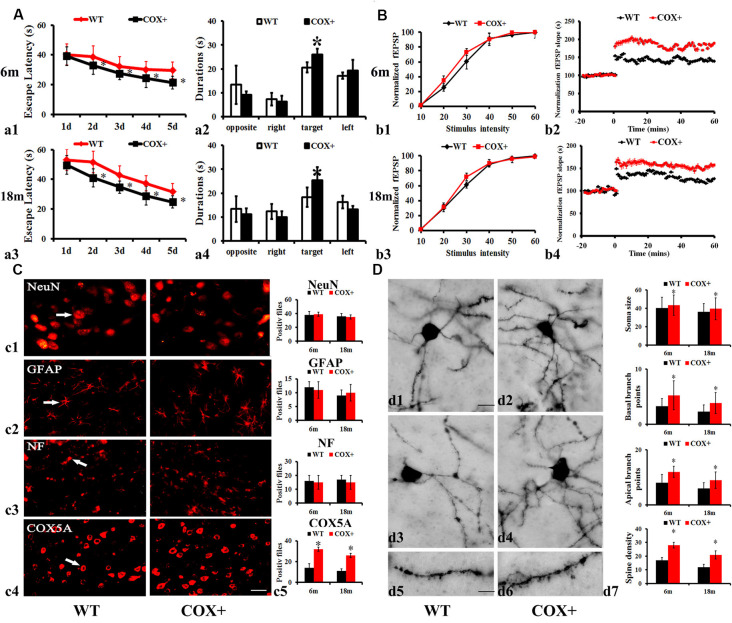
Effects of COX5A overexpression in mice. **(A)** Average escape latency **(a1,a3)** and the duration in the target quadrant** (a2,a4)** evaluated by the Morris Water Maze (MWM) test in 6-months-old **(a1,a2)** and 18-months-old **(a3,a4)** COX5A-UP transgenic (Tg) and age-matched WT mice. Opposite, the opposite quadrant; right, the right quadrant; target, the target quadrant; left, the left quadrant. * vs. WT mice, *P* < 0.05. Values plotted to represent the mean ± SD (*n* = 10). **(B)** Synaptic LTP of the hippocampus in COX5A-UP Tg mice and WT mice. Values plotted to represent the mean ± SD (*n* = 5). * vs. WT mice, *P* < 0.05. **(C)** Morphologic changes in COX5A-UP Tg mice. **(c1–c4**) NeuN, GFAP, NF, and COX5A, respectively; Magnification: 200×, Scale bar: 10 μm. **(D)** Representative images of neurons with Golgi stains in 6-months-old **(d1,d5)** and 18-months-old **(d3)** WT mice, as well as in 6-months-old **(d2,d6)** and 18-months-old **(d4)** COX5A-UP Tg mice. The quantification of changes in dendritic cells is shown in **(d7)**. Magnification: **(d1–d4)**: 200×, Scale bar, 10 μm; **(d5)** and **(d6)**: 400×; Scale bar, 5 μm. COX+, COX5A-UP Tg mice; WT, wild type mice.

On the first test day of the hidden platform test, the average escape latency was not affected by genotype (COX5A-UP Tg: *F* = 0.012, *P* = 0.854; WT: *F* = 0.025, *P* = 0.925), nor age group (COX5A-UP Tg: *F* = 0.058, *P* = 0.924; WT: *F* = 0.018, *P* = 0.685). Moreover, repeated-measures ANOVA (RM ANOVA) revealed that the escape latency of the hidden platform progressively decreased with remaining training days in all groups (Supplementary Table S2). A main effect of genotype was observed for escape latency in 6-months-old COX5A-UP Tg mice (*F* = 30.15, *P* < 0.01) and 18-months-old COX5A-UP Tg mice (*F* = 39.51, *P* < 0.01). Age was another main factor (Supplementary Table S2). Taken together, the data indicated an age-dependent behavioral impairment in both WT and Tg mice, while COX5A overexpression served as a cognitive enhancer. Compared with the age-matched control WT mice, COX5A-UP Tg mice needed less time to find the hidden platform underwater on day 2–5 of the test (Supplementary Table S2; Figures 3a1,a3). At the end of the hidden platform test, the platform was removed for the probe trial, and all mice were allowed to swim for 60 s to find the platform. COX5A-UP Tg mice showed a significant preference for the target quadrant when compared with age-matched WT mice (6-months-old: Figure 3a2, *F* = 31.23, *P* = 0.00014, *P* < 0.05; 18-months-old: Figure 3a4, *F* = 29.01, *P* = 0.00025, *P* < 0.05).

Moreover, we also analyzed the average speed and the distance to the target of WT mice and COX5A transgenic mice during the probe trial. The results were shown in Supplementary Figure S6. The mean swimming speed of 18-months-old WT mice significantly decreased when compared with that of the 6-months-old WT ones (*P* < 0.05). The distance in the probe trial showed no significant difference between 18-months-old and 6-months-old WT mice (*P* > 0.05). However, the statistical significance was found in the mean swimming speed and distance to target between the 18-months-old WT mice and the age-matched COX5A-UP transgenic (Tg) mice (*P* < 0.05). Given that muscle has lots of mitochondria and muscle function can be affected by age as well, COX5A overexpression compensates the athletic ability losses caused by age. Although, COX5A overexpression increased the swim speed in the MWM test, the up-regulation in COX5A also explained the differences in learning. In Supplementary Figure S6, the results showed that during the probe trial, COX5A overexpressed mice had higher percentages of time in the target quadrant, percentages of their path in the target quadrant, and the number of platform crossings when compared with that of the age-matched WT ones (*P* < 0.05). Despite the compensations in the athletic ability losses caused by age, COX5A up-regulation also induced the improvement in learning and memory in 18-month aged mice.

Hippocampal synaptic plasticity indicated by LTP showed a significant improvement in COX5A-UP Tg mice. RM ANOVA revealed a main genotype effect (*F* = 25.24, *P* = 0.00017, *P* < 0.05) and age (*F* = 21.36, *P* = 0.00041, *P* < 0.05) (Supplementary Table S3). Initial characterization of the hippocampal section of COX5A-UP Tg mice revealed no obvious deficits in the basic properties of CA1 synapses [*P* = 0.08, *N* = 5, *n* = 5 (*N* = 5 mice per group; *n* = 5 sections per mouse), Figures 3b1,b3]. The input-output curves in COX5A-UP Tg and WT mice were virtually indistinguishable (Figures 3b1,b3). A high frequency, tetanic stimulation train (HFS, 100 Hz, 1 s was used to induce LTP. HFS produced a robust LTP in COX5A-UP Tg mice when compared to that of WT mice (6-months-old: Figure 3b2, *P* = 0.00021, *P* < 0.05, Figure 3b2; 18-months-old: Figure 3b4, *P* = 0.00036, *P* < 0.05, Figure 3b4). Even though the results are negative, they also have implications for us. There might be molecular neuronal plasticity that occurred before distinguishable morphological changes were detected. The present study just observed the possible morphological changes at 18 months. Further investigations involved in older transgenic mice are required to evaluate the potential changes in macroscopic alteration.

The immunoreactive (IR) profiles of COX5A were scattered throughout the cortex and hippocampus of both Tg and WT mice. Also, COX5A signals were present in neuronal membranes, the cytoplasm, and processes, but not in the nucleus. However, the numbers of NeuN (Figure 3c, *P* = 0.061, *n* = 5), GFAP (Figure 3c1, *P* = 0.072, *n* = 5), and NF-100 (Figure 3c2, *P* = 0.083, *n* = 5) IR profiles in COX5A-Tg mice showed no significant differences when compared with age-matched non-Tg control mice (Figure 3c3).

The recovery of hippocampal CA1 dendrites in COX5A-Tg mice was investigated in the present study. In CA1 dendrites, the basal region of the dendritic branches showed significant differences between COX5A-UP Tg and WT mice, as shown in representative photomicrographs of hippocampal CA1 neurons in Figure 3D. In WT mice, there was a decrease in spine density with age. Images represent the dendritic pattern of each experimental group. An increase in apical/basal dendritic branches was observed in Tg mice when compared with age-matched WT mice (Figures 3d1–7, *P* = 0.0001, *P* < 0.05, *N* = 5, *n* = 5). Next, we evaluated the average dendritic spine number per 100 μm of dendrites. The data showed that COX5A-UP Tg mice exhibited a significant restoration in the dendritic spine when compared with matched WT mice (Figures 3d2,d4,d6,d7; *P* = 0.00072, *P* < 0.05, *n* = 5).

### Changes in Gene and Protein Expression of BDNF-Related Signaling Pathways in Tg Mice

**Figure 4:**
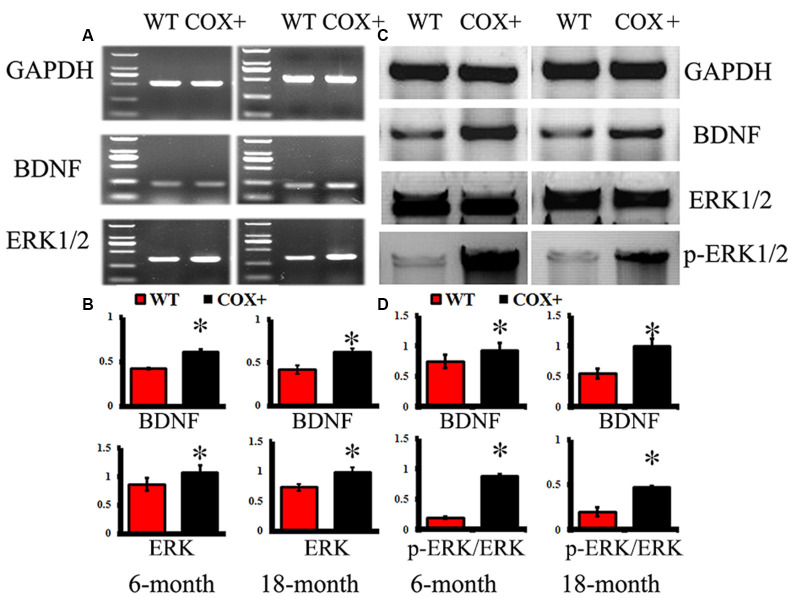
Effects of COX5A on the expression of BDNF and ERK1/2 signaling in mice. WT, wild type mice; COX+, COX5A-UP transgenic (Tg) mice. Panel **(A)** shows representative products electrophoresed on a 1% agarose gel. Lane 1: DNA marker DL 2,000 (from top to bottom: 2000 bp, 1000 bp, 750 bp, 500 bp, 250 bp, 100 bp). Genes included BDNF and ERK1/2. GAPDH served as a control. **(B)** Quantification of the expression of different genes. The mRNA levels of BDNF and ERK1/2 were significantly up-regulated in COX5A-UP Tg mice. * vs. WT mice (*P* < 0.05). The changes in protein expression in BDNF-related signaling pathways were determined by Western blot analysis (WB). **(C)** Representative lanes of proteins (BDNF, ERK1/2, and p-ERK1/2) determined by WB. **(D)** Quantification of the expression of different proteins, including BDNF, ERK1/2, and p-ERK1/2. Values plotted to represent the mean ± SD (*n* = 10). * vs. WT mice, *P* < 0.05.

In the hippocampus of COX5A-UP Tg mice, mRNA levels of BDNF and ERK1/2 were significantly up-regulated at 6 and 18 months when compared with WT mice (Figures 4A,B, *P* = 0.000019, *P* < 0.05, *n* = 10). The relative abundance of BDNF phosphorylated ERK1/2 (p-ERK1/2), and total ERK1/2 (ERK1/2) was determined by Western blot analysis followed by densitometry. Consistent with the RT-PCR results, levels of BDNF (*P* = 0.00003, *P* < 0.05, *n* = 10) and phosphorylated ERK1/2 (*P* = 0.00022, *P* < 0.05, *n* = 10) in the hippocampus of COX5A-UP Tg mice were significantly increased when compared with age-matched WT mice (Figures 4C,D). Our results showed that the protein levels of BDNF, ERK1/2, and p-ERK1/2 were significantly up-regulated in COX5A+ Tg mice vs. WT mice at both 6-months-old (*P* = 0.00011, *P* = 0.000012, *P* = 0.00023, respectively, *P* < 0.05, *n* = 10) and 18-months-old mice (*P* = 0.00003, *P* = 0.000018, *P* = 0.000011, respectively, *P* < 0.05, *n* = 10). The ratios of p-ERK1/2/ ERK1/2 were calculated, and in 6-months-old mice, the ratio of p-ERK1/2/ ERK1/2 in 1~7COX5A-UP Tg mice was higher when compared to that in WT mice (COX5A-UP Tg vs. WT, 1.13 ± 0.05 vs. 0.87 ± 0.08, *P* = 0.000001, *P* < 0.05, *n* = 10). In 18-months-old mice, the ratio of p-ERK1/2/ ERK1/2 in COX5A-UP Tg mice was significantly increased when compared with WT mice (COX5A-UP Tg vs. WT, 0.97 ± 0.02 vs. 0.69 ± 0.05, *P* = 0.00029, *P* < 0.05, *n* = 10, Figures 4C,D).

### COX5A Regulates Recognition *via* a BDNF-Dependent Signaling Pathway *in vivo*

To determine whether COX5A could regulate BDNF and affect cognitive function, four heterozygous Tg offspring of the BDNF-KD line was developed. The identification of BDNF-KD mice is described in detail in the Supplementary Materials. In brief, COX5A-UP/BDNF-KD Tg mice were established by cross-breeding with both Founder 39 (BDNF-KD) and Founder 35 (COX5A-UP). Double-Tg mice were identified by PCR (Supplementary Figure S3). To determine whether COX5A could regulate the BDNF signaling pathway, the following was evaluated: (i) changes in CcO activity in hippocampal mitochondria (Supplementary Figure S3); (ii) alterations in ATP levels in hippocampal mitochondria (Supplementary Figure S3); (iii) cognitive function using the MWM test; (iv) synaptic LTP analysis in the hippocampus; and (v) quantified changes in hippocampal CA1 dendrites.

**Figure 5:**
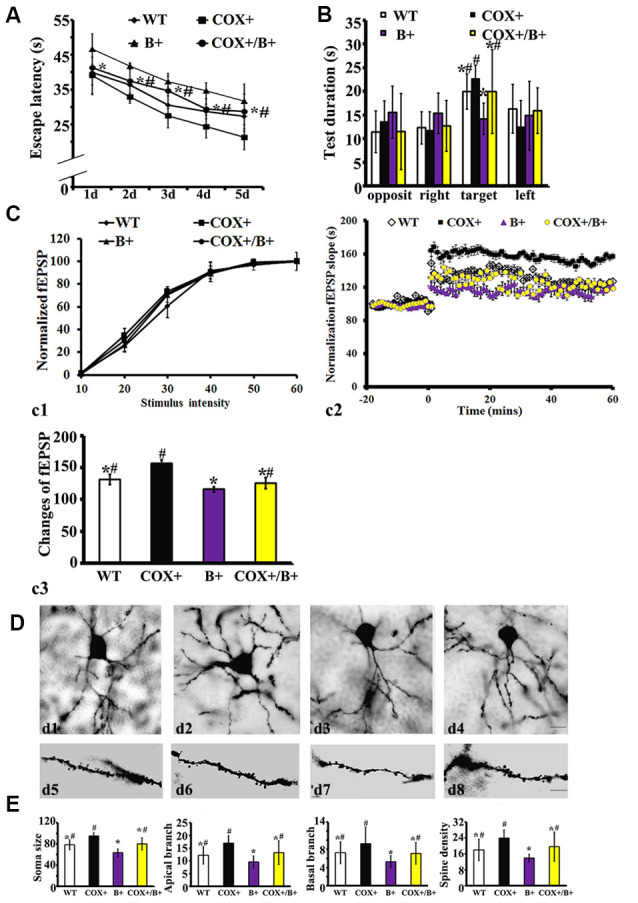
Effects of COX5A exerted by a BDNF-dependent pathway *in vivo*. WT, wild type mice; COX+, COX5A-UP Tg mice; B+, BDNF-KD Tg mice; COX+/B+, COX5A-UP/BDNF-KD Tg mice. **(A)** and **(B)** Results of the Morris Water Maze (MWM) test in mice with different genotypes. Values plotted to represent the mean ± SD (*n* = 10). * vs. COX5A-UP Tg mice, *P* < 0.05; ^#^vs. BDNF-KD Tg mice, *P* < 0.05. **(C)** Synaptic LTP of the hippocampus in COX5A-UP, BDNF-KD, and COX5A-UP/ BDNF-KD Tg mice. **(D)** Representative images of neurons with Golgi stains in WT **(d1,d5)** COX5A-UP **(d2,d6)** BDNF-KD **(d3,d7)** and COX5A-UP/BDNF-KD Tg mice **(d4,d8)**. **(E)** Quantification of hippocampal CA1 dendrites and neurons among genotypic Tg mice and WT mice. Values plotted to represent the mean ± SD (*n* = 5). * vs. COX5A-UP Tg mice, *P* < 0.05; ^#^vs. BDNF-KD Tg mice, *P* < 0.05. Magnification, (**d1–d4**): 200×, Scale bar, 10 μm; d5–d8, 400×; Scale bar, 5 μm.

In the hidden platform test, the average escape latency was affected by genotype (RM ANOVA). There was a significant effect of genotype on the escape latency in COX5A-UP Tg mice (*F* = 38.25, *P* < 0.01), BDNF-KD Tg mice (*F* = 35.27, *P* < 0.01) and COX5A-UP/BDNF-KD double-Tg mice (*F* = 29.14, *P* < 0.01). When compared with that of control WT mice, the escape latency of BDNF-KD Tg mice was significantly delayed during the entire experimental period (*P* = 0.000004, *P* < 0.05, *n* = 10). In the spatial probe trial, BDNF-KD Tg mice showed significant hesitation for the target quadrant when compared with WT mice (*P* = 0.0007, *P* < 0.05, *n* = 10). Moreover, double-Tg mice exhibited a delay in the average escape latency (*P* = 0.000, *P* < 0.05, *n* = 10) and significant hesitation for the target quadrant (*P* = 0.000, *P* < 0.05, *n* = 10) when compared with that of COX5A-UP Tg mice, respectively. When compared with BDNF-KD mice, double-Tg mice showed significant improvement in the spatial test (Supplementary Table S4; Figures 5A,B).

In hippocampal synaptic plasticity analysis, RM ANOVA revealed a significant genotype effect (Supplementary Material, Table S5). Synaptic LTP evaluation in the hippocampus confirmed that the input-output curves between the different genotypes of Tg and WT mice were virtually indistinguishable (Figure 5c1; *P* = 0.12, *P* > 0.05). As demonstrated previously, HSF induced a robust fEPSP slope in COX5A-UP Tg mice when compared with WT mice, BDNF-KD, and COX5A-UP/BDNF-KD Tg mice (COX5A-UP vs. WT, *P* = 0.00002, *P* < 0.05; COX5A-UP vs. BDNF-KD, *P* = 0.000033, *P* < 0.05; COX5A-UP vs. COX5A-UP/BDNF-KD, *P* = 0.000012, *P* < 0.05, *n* = 5). However, the slope was significantly subdued in BDNF-KD Tg mice (COX5A-UP vs. BDNF-KD, *P* = 0.000003, *P* < 0.05, *n* = 5) and exhibited a partial recovery in COX5A-UP/BDNF-KD Tg mice (Figures 5c2,c3, BDNF-KD vs. COX5A-UP/ BDNF-KD, *P* = 0.000000, *P* < 0.05, *n* = 5).

Quantification of hippocampal CA1 dendrites showed that the apical (*P* = 0.000013, *P* < 0.05, *n* = 5), and basal dendritic arbors (*P* = 0.000048, *P* < 0.05, *n* = 5), and the average dendritic spine number (per 100 μm, *P* = 0.0006, *P* < 0.05, *n* = 5) were significantly decreased in BDNF-KD mice when compared with COX5A-UP/BDNF-KD Tg mice (Figures 5D,E). Representative photomicrographs of hippocampal CA1 neurons from BDNF-KD and COX5A-UP/BDNF-KD Tg mice are presented in Figures 5d3,d4 and Figures 5d7,d8, respectively.

### COX5A Promotes Neurite Outgrowth in Hippocampal Neurons *via* a BDNF-Dependent Pathway *in vitro*

**Figure 6:**
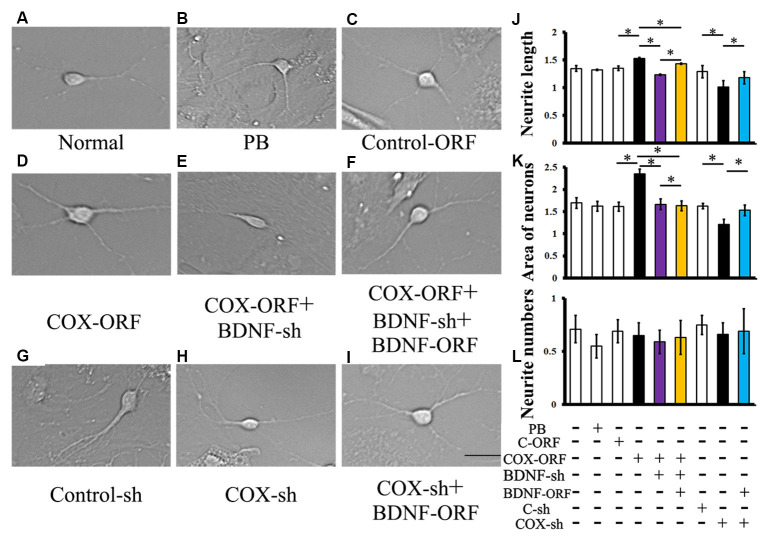
Effects of COX5A *via* a BDNF-dependent pathway *in vitro*. **(A–I)** Morphology and axonal length of hippocampal neurons transfected with different constructs. **(J–L)** Quantification of neurite extensions. Quantification of neurite length **(J)**, the areas of cultured neurons **(K)**, and the number of neurites **(L)** after introduction with COX5A-ORF, COX5A-shRNA, BDNF-ORF, and/or BDNF-shRNA, respectively. Values plotted to represent the mean ± SD. *P < 0.05, C-ORF, control-ORF; COX-ORF, COX5A-ORF; C-sh, control-shRNA; COX-sh, COX5A-shRNA, BDNF-sh, BDNF-shRNA. Magnification **(A–I)**, 400×; Scale bar, 5 μm.

Introduction of COX5A-ORF together with BDNF-shRNA into cultured hippocampal neurons significantly decreased neurite extensions (*P* = 0.0003, *P* < 0.05, *n* = 5) and neuron area (*P* = 0.000011, *P* < 0.05, *n* = 5) when compared with COX5A-ORF treatment alone. However, administration with BDNF-ORF after treatment with both COX5A-ORF and BDNF-shRNA rescued hippocampal cells by restoring both neurite extensions (*P* = 0.000012, *P* < 0.05, *n* = 5) and the neuron area (*P* = 0.000001, *P* < 0.05, *n* = 5; Figure 6).

Conversely, transduction with both COX5A-shRNA and BDNF-ORF increased both neurite extensions (*P* = 0.000009, *P* < 0.05, *n* = 5) and the neuron area (*P* = 0.000014, *P* < 0.05, *n* = 5) when compared with COX5A-shRNA-treated neurons (Figures 6B,D). However, administration with COX5A-ORF, COX5A-shRNA, BDNF-ORF, or BDNF-shRNA did not affect the neurite number (Figure 6L).

Moreover, conduction with a specific ERK1/2 inhibitor, PD98059, in COX5A-ORF or BDNF-ORF-treated neurons significantly attenuated the effects on neurite extensions induced by COX5A up-regulation. Also, pretreatment with PD98059 neutralized the dendritic restoration induced by BDNF overexpression (BDNF-ORF) after COX5A-shRNA transfection (Supplementary Figure S5).

## Discussion

To explore the potential effects of COX5A on aging, the generation of animal models using genetic engineering was applied in this study. This transgenic model leads to stable genomic integration *in vitro* or *in vivo* with extremely high efficiency (Aguzzi et al., 1994). We successfully established COX5A up-regulated mice. To our knowledge, this is the first study to report that COX5A up-regulated by 51% results in a remarkable improvement in hippocampal-dependent spatial recognition memory. This improvement corresponded to an increase in dendritic branching points in the CA1 region, mitochondrial CcO activity, and ATP concentration in the hippocampus. Consistently, these changes up-regulated the expression of BDNF and ERK1/2. Moreover, double Tg mice with COX5A overexpression and BDNF knockdown confirmed both *in vivo* and *in vitro* that COX5A exerted its cognitive neuroprotective effects *via* BDNF. Administration of an ERK1/2 inhibitor attenuated the promotion of neurite extensions that were induced by COX5A or BDNF. Therefore, the present findings provide crucial evidence to elucidate the role of COX5A and its related signaling pathway in the aging process, which may be useful to screen novel targets for the treatment of aging-related impairments in cognition.

COX, located deeply in the mitochondrial inner membrane, is the terminal enzyme of the respiratory chain (Bai et al., 2020). During catalysis, the consumption of protons inside the mitochondrial matrix, together with the translocation of other protons, contributes to the growth of the electrochemical potential gradient utilized to synthesize ATP (Capaldi et al., 1990; Poyton and McEwen, 1996). In mammals, COX is composed of 13 subunits (three mitochondrial-encoded subunits and 10 nuclear-encoded subunits), some of which occur as tissue-specific isoforms (Grossman and Lomax, 1997). COX5A is one of the three mitochondrial-encoded subunits, which constitutes the catalytic center of the enzyme. It has previously been demonstrated that COX5A can cross-link with subunits I, II, III and VII, and subunit IV with subunits VI and VII, suggesting near-neighbor relationships among the first and second group of subunits (Briggs and Capaldi, 1977). Moreover, COX contributed to neuronal plasticity after traumatic brain injury (TBI), and it has been reported that TBI decreased the expression of sphingosine kinase 2 (SphK2), the complex of neutral ceramidase and COX subunit 1(COX-1), thereby further influencing mitochondrial function (Novgorodov et al., 2014). Although it is known that COX5A is essential for modulating cell survival and activity in an altered metabolism induced by either physiology or a pathological condition (Chen et al., 2012; Freije et al., 2012), and it serves as a metabolic marker (Albarracin et al., 2013), few studies have investigated the roles of COX5A in aging. The present study is the first to demonstrate that COX5A expression was decreased in the hippocampus of aged SAMP8 mice. Therefore, COX5A may correlate with the electron transport chain, as was indicated by previous studies, and may be associated with aging and age-related diseases (Strazielle et al., 2009; Atamna and Kumar, 2010). Taken together, the decrease of COX5A in aged SAMP8 mice suggested that COX5A might contribute to age-related memory impairment.

*In vivo* observations showed that up-regulation of COX5A by 51% resulted in the following: (i) improvement in hippocampal-dependent spatial recognition memory; (ii) a significant increase in LTP of the fEPSP slope; (iii) an increase in branching points and spine density of CA1 dendrites; (iv) and a restoration of mitochondrial CcO activity and ATP concentration. These results indicated that COX5A plays a crucial role in learning and memory and strongly suggested that COX5A is an important molecule in aging. The mechanisms underlying cognitive improvement in COX5A overexpressing mice may be related to the increases in spine density in hippocampal CA1, which can up-regulate the synaptic excitability of an entire dendrite (Moulin et al., 2019). It has previously been suggested that the activation of spiny neurons may depend as much on the density as on the number of active synapses (Briones et al., 2018). The *in vitro* observations support the findings *in vivo*, in which COX5A is vital for neurite growth. Taken together, COX5A may regulate BDNF/ERK1/2 expression, as indicated by RT-PCR findings.

It is known that BDNF, a neurotrophic factor (NTF), is a small, versatile protein that can maintain neuronal survival, axonal guidance, and cell morphology (von Bohlen Und Halbach and von Bohlen Und Halbach, 2018; Luo et al., 2020). BDNF plays key roles in cognition and memory formation, which has been well studied (Luo et al., 2020). Interestingly, previous studies have demonstrated that aging is characterized by a significant decline in NTF levels, which often contributed to the onset of severe age-associated pathologies (Tapia-Arancibia et al., 2008). As a vital molecule, BDNF has many critical roles in maintaining neuronal survival and regulating plasticity, memory, and learning. BDNF is also involved in the pathogenesis of AD (Tapia-Arancibia et al., 2008; Chen et al., 2019). Changes in BDNF expression levels and distribution, as well in its receptor, tyrosine kinase type 2 (TrkB), have also been reported in patients and animal models of AD (Schulte-Herbruggen et al., 2008). Because the role of BDNF in aging and AD is clear, we, therefore, did not investigate the effect of BDNF on aging. Instead, in this study, we investigated whether COX5A improved cognitive function, which is dependent on BDNF/ERK1/2 signaling. Down-regulation of BDNF levels both *in vivo* and *in vitro* markedly attenuated the effects of COX5A overexpression. These findings confirmed that COX5A plays a role in cognition and requires the BDNF signaling pathway. This is a novel finding that may explain the mechanisms of COX5A in aging-related diseases.

Our data showed that *in vivo* up-regulation of COX5A in COX5A-UP Tg mice resulted in the up-regulation of NTFs, including BDNF and downstream signaling molecules, such as ERK1/2. Moreover, our *in vitro* data also showed that the expression of BDNF and ERK1/2 were reduced after interfering with COX5A. Importantly, a 55% reduction in BDNF expression in BDNF-KD mice caused impairment in spatial recognition and memory and decreased dendritic branching points in hippocampal CA1, even in situations when COX5A was overexpressed. Partial recovery was observed in the BDNF rescue setting. Also, confocal images demonstrated that COX5A and BDNF colocalized in hippocampal neurons (Supplementary Figure S4). Blocking the ERK1/2 signaling pathway by using PD98059 attenuated neurite promotion elicited by COX5A or BDNF (Supplementary Figure S5). Together, these results demonstrated that COX5A exerted its neuroprotective effects in cognition *via* a BDNF-regulated ERK1/2 signaling pathway. It has previously been shown that neurotrophic BDNF deficiency begins in the early stages of age-related diseases, including AD, and eventually causes a decrease in learning and memory signal transduction, such as the MAPK/ERK/CREB pathway (Liu et al., 2013; Zhao et al., 2013). Our findings showed that BDNF levels and phosphorylated ERK1/2 expression in the hippocampus were suppressed in aged WT mice. Amazingly, genetic COX5A up-regulation induced elevated BDNF levels and restored phosphorylated ERK1/2 expression in the hippocampus of COX5A-UP Tg mice. Learning and memory are controlled at the molecular level by several major signaling pathways in the brain, one of which is MAPK (Ryu and Lee, 2016). In the MAPK family, ERK1/2 converts a signal into a transcription factor, which subsequently binds to the promoter region of many genes associated with memory and synaptic plasticity (Pham et al., 1999; Barco et al., 2003). BDNF is one of the many effectors of ERK phosphorylation regulation and participates in learning and memory processes (Ko et al., 2017; Jin et al., 2019). ERK/CREB/BDNF can be greatly suppressed by sublethal Aβ treatment in cortical neuronal loss (Tong et al., 2004). In the present study, phosphorylation of ERK1/2 and BDNF proteins was reduced in aged WT mouse hippocampi. COX5A overexpression rescued the repression of the BDNF/ERK1/2 pathway, which is in line with the results presented in previous studies, thereby indicating that the BDNF/ERK1/2 pathway is a molecular therapeutic pathway in the treatment of cognitive impairment in Tg AD mice (Liu et al., 2013; Hou et al., 2010). Thus, coupling cognitive improvement, the results obtained in the present study revealed that COX5A might affect the modulation of synaptic transduction of BDNF/ERK1/2 in hippocampal-dependent cognitive function. Otherwise, the regulation of the BDNF/ERK1/2 pathway has also been observed in cognitive impairment induced by other cerebral diseases, such as TBI, which causes neurodegeneration due to mechanical impact from external forces (Gardner and Yaffe, 2015). BDNF treatment post-TBI in mice induced significant improvement in cognitive impairment when compared with TBI mice that did not receive treatment (Khalin et al., 2016). Moreover, exercise before TBI injury could increase the expression of COX I, II, III, repair the CcO activity and rescue ATP levels in mitochondria to improve cognitive function (Gu et al., 2014). Therefore, we provide a novel way to explore the potential mechanism among cognitive impairment and plasticity during brain diseases.

## Conclusions

Our study first supplies supporting evidence that COX5A is a significant and treatable component of cognitive impairment induced by brain senescence-associated diseases. In the present study, we provide novel crucial evidence that COX5A, *via* BDNF activating downstream molecules, such as ERK1/2, plays a very important role in aging-related cognitive deterioration in the mouse. Our findings may offer a potential strategy for the treatment of aging-related diseases in clinical trials.

## Data Availability Statement

All datasets generated for this study are included in the article/Supplementary Material.

## Ethics Statement

The animal study was reviewed and approved by the ethics committee of the Kunming Medical University in Yunnan, China (permit number: km-edw-2013118).

## Author Contributions

Y-BX, T-HW, and JZ designed the study, performed analyses, and prepared the manuscript. Y-BX, SL, RL, X-YW, YZ, B-TL, Z-CX, and JZ conducted mouse experiments, lentiviral vector construct, production and transductions, cell culture, cell transfection, and cell treatment. Y-BX, RL, X-YW, YZ, B-TL, and T-HW performed RNA labeling and array hybridization, mitochondria COX activity assay and ATP measurement, Western blotting, RT-PCR, immunofluorescence detection and analysis. Y-BX, X-YW, YZ, B-TL, Z-CX, L-FZ, and JZ conducted the electrophysiology evaluation, Golgi stains, and behavior test. Y-BX, B-TL, Z-CX, L-FZ, T-HW, and JZ finished the BDNF rescue setting and statistical analyses. All authors were substantially involved in the research, acquisition of data, analysis, and manuscript preparation and have read and approved the final submitted manuscript.

## Conflict of Interest

The authors declare that the research was conducted in the absence of any commercial or financial relationships that could be construed as a potential conflict of interest.
