# Supplementary material for: COX5A Plays a Vital Role in Memory Impairment Associated With Brain Aging via the BDNF/ERK1/2 Signaling Pathway
Source: Front Aging Neurosci. 2020 Jul 10;12:215. doi: 10.3389/fnagi.2020.00215 (PMC7365906; doi:10.3389/fnagi.2020.00215)
Supplement: Supplementary file 1 [file Data_Sheet_1.docx]

***Supplementary Material***

# Material and Methods

## Immunofluorescence analysis

After intraperitoneal injection with 3.6% chloral hydrate (1 ml/100 g), mice were perfused with 150 ml of cold phosphate-buffered saline (PBS) for 5 min, followed by 150 ml of 4% paraformaldehyde solution for 30 min. The hippocampus from mice in each group was harvested, post-fixed for 6-12 h with paraformaldehyde, then immersed overnight in 0.1 M PBS, containing 20% sucrose until the specimen sank to the bottom of the flask. Sections of 20-µm thickness were cut using a freezing microtome (Leica CM1900, Germany) and collected in a 24-well plate. Frozen sections were washed three times with 0.01 M PBS for 5 min each, and prepared for immunofluorescence analysis. In brief, sections were blocked for 30 min at 37°C with blocking solution containing 5% goat serum and 0.3% Triton X-100. Then, sections were incubated overnight at 4°C with primary antibodies, including COX5A (1:200, Santa Cruz), NeuN (1:500, Chemicon), GFAP (1:500, Santa Cruz), NF-100 (1:4,000, Immunostar), and BDNF (1:1,000, Chemicon). Subsequently, sections were washed three times with 0.01 M PBS for 5 min each, and incubated for 2 h at 37°C with fluorescently-labeled secondary antibodies (Abbkine, CA, USA). To determine the effects of COX5A on the nervous system, immunolabeling with specific markers (NeuN, GFAP, and NF) was implemented. Hippocampal COX5A co-localization studies with neurons and BDNF were also performed. Antifade Mounting Medium (Beyotime, Shanghai, China), containing DAPI was used to counterstain the nuclei. Images were acquired using a fluorescent microscope (LEICA DMI6000B, Germany)

## Animal models

COX5A-UP, BDNF-KD, and COX5A-UP/BDNF-KD transgenic (Tg) mice on a C57BL/6J genetic background were established by our collaborators at The Institute of Laboratory Animal Science (Chinese Academy of Medical Sciences & Comparative Medicine Centre, Peking Union Medical College, Beijing, China). Briefly, full-length ORF cDNA clones for the COX5A gene ((Mus musculus), GeneID: 12858) were purchased from Origene (Rockville, MD, USA). Subsequently, ORFs with restriction sites were obtained by PCR and directionally cloned into plasmid pMD-18T simple vectors (purchased from Takara, Osaka, Japan). Products were determined by the Sanger's protocol (Kim et al., 2020). The cytomegalovirus immediate early gene promoter was inserted downstream in the COX5A gene using a newly created EcoRI/XhoI restriction site (Supplementary Figure, Fig. S2). Next, the transgene was isolated from the cloning plasmid by PvuI digestion, purified by CsCl gradient centrifugation, and microinjected into fertilized mouse eggs (F1[C57BL/6xCBA/J] x F1[C57BL/ 6xCBA/J]).

The silence expression vector for BDNF and the reconstruction plasmid (Supplementary Figure, Fig. S3) were designed by and purchased from Invitrogen (Carlsbad, CA, USA). The constructed recombinant plasmid was transferred into 293T cells, and transformants were screened and identified by PCR and restriction analysis. Subsequently, transgenes were isolated from the cloning plasmid by Avr II digestion, purified by CsCl gradient centrifugation, and microinjected into fertilized mouse eggs (F1[C57BL/6xCBA/J] x F1[C57BL/6xCBA / J]).

Tg mice were mated with either non-Tg partners to maintain heterozygosity of the transgene or Tg partners to generate homozygous Tg offspring. In the latter case, Tg male mice were test-mated with two wild-type female mice, and their offspring (15-20 mice) were analyzed by PCR. Male mice that produced exclusively Tg offspring were considered homozygous for the transgene.

Inserted fragments (COX5A, 386bp; BDNF, 489bp) were identified by PCR. For COX5A up-regulated lines, the following primers were used: sense, 5′-GTCAATGGGTGGAGTATTTACG -3′; antisense, 5′-GCTTATATAGACCTCC-

CACCGT-3′. For BDNF down-regulated lines, the following primers were used: sense, 5′-TGTGACAGTATTAGCGAGTGGGT -3′; antisense, 5′-TACGATTGGGT-

AGTAGTTCGGCATT-3′. Briefly, PCR was performed using the PCR Master Mix Kit (Fermentals Company, USA) for 35 cycles as follows: denaturation at 94°C for 30 seconds, annealing at 58°C for 30 seconds, and extension at 72°C for 30 seconds. Then, RT-PCR products were electrophoresed using a 1% agarose gel stained with ethidium bromide, and visualized using an ultra violet gel imager (BIO-GEL, BIO-RAD). Image analysis was performed using I mager J 6.0 software (L IVE Science, Massachusetts, USA). Homozygous Tg offspring with up-regulated COX5A were mated with down-regulated BDNF to generate double Tg mice. Tg offspring were identified by PCR as described above. Mice with both inserted fragments were considered homozygous for the transgenes.

COX5A-UP, BDNF-KD, COX5A-UP/BDNF-KD Tg mice and age-matched, non-Tg littermates (wild-type, WT mice) were used. Mice were housed with free access to food and water, and were housed in an environment with a 12-h light/dark cycle. Animal use and care were in accordance with the Guide for the Care and Use of Laboratory Animals as published by the US National Institutes of Health (NIH Publication No. 85-23, revised 1996).

# **Results**

## Subcellular localization of COX5A and BDNF in the hippocampus

To determine the roles of COX5A and BDNF in age-related defects in cognition, the hippocampal subcellular localization in mice was first identified by co- immunofluorescent staining of mitochondrial COX5A and NeuN as well as of COX5A and BDNF. Neuronal labeling with COX5A/NeuN (Supplementary Figure, Fig. S4-a, b, c and d) and COX5A/BDNF showed that COX5A co-localized with BDNF and was symmetrically distributed within the cytoplasm of hippocampal neurons in mice (Supplementary Figure, Fig. S4-e, f, g and h).

## Effects of COX5A over-expression *in vivo*

Supplementary Figure, 3-a and b, shows the illustration of pcDNA3.1 (+) for COX5A-UP Tg mice, which comprised of 5428 nucleotides. Locations were as follows: of the CMV promoter: bases 232-819, T7 promoter/priming site: bases 863-882, multiple cloning site: bases 895-1010, pcDNA3.1/BGH reverse priming site: bases 1022-1039, bovine growth hormone (BGH) polyadenylation sequence: bases 1028-1252, f1 origin: bases 1298-1726, SV-40 early promoter and origin: bases 1731-2074, neomycin resistance gene: bases 2136-2930, SV40 early polyadenylation signal: bases 3104-3234, pUC origin: bases 3617-4287 (complementary strand), ampicillin resistance gene (bla): bases 4432-5428 (complementary strand), open reading frame (ORF): bases 4432-5292 (complementary strand); ribosome binding site: bases 5300-5304 (complementary strand); bla promoter (P3): bases 5327-5333 (complementary strand). Genotypes of Tg mice were by PCR and determined the data showed that five heterozygous Tg offspring of COX5A-UP lines were obtained. Among them, only four produced offspring. These were designated as Founder 35, Founder 22, Founder 26, and Founder 28. Tg mice with an inserted fragment, as identified by PCR, were considered positive Tg (Supplementary Figure, Fig.S2-c).

Then, expressional changes of COX5A in the hippocampus of Tg mice with different genotypes were indicated by RT-PCR and Western blot analysis. Results of Western

blot analysis showed that COX5A expression in the hippocampus of Tg mice of the four genotypes, were up-regulated by 51.62%, 30.09%, 20.61%, and 17.57% in Founder 35, Founder 22, Founder 26, and Founder 28, respectively (Supplementary Figure, Fig. S2-e). The rate of protein up-regulation was calculated as follows: Rate of protein up-regulation= O.D. (Founder-WT) / O.D. WT × 100%. The results obtained by RT-PCR (Supplementary Figure, Fig. S2-d) were consistent with the data obtained by Western blot analysis. Founder 35 was chosen as the target line as it showed the highest value.

Cytochrome c oxidase (CcO) activity analysis of hippocampal mitochondria revealed that in the 18-month-old group, CcO activity (nmol/mg of mitochondrial protein per minute) was progressively reduced in both of WT and Tg mice when compared with that in 6-month-old mice (18-month-old WT vs 6-month-old WT, P=0.00032, P<0.05; 18-month-old Tg vs 6-month-old Tg, P=0.00032, P<0.05; n=10). As shown in Supplementary Figure S3f, significantly decreased CcO activity was observed in hippocampal mitochondria of WT mice at both 6 months (P=0.00000, P<0.05, n=10) and 18 months of age (P=0.000021, P<0.05, n=10) when compared to that of COX5A-UP Tg. Notably, levels of CcO activity in mitochondria from COX5A Tg mice were restored comparable to those in age-matched WT mice (Supplementary Figure, Fig. S2-f, P=0.00001, P< 0.05, n=10).

The ATP content measured in mitochondria from both WT and COX5A-UP Tg mice showed an age-related decline from 6 month to 18 months (Supplementary Figure, Fig. S2-g, P=0.00032, P<0.05). However, the age-related reduction in ATP content was partially restored in COX5A-UP Tg mice, when compared to that in age-matched WT mice (Supplementary Figure, Fig. S2-g, P=0.00011, P < 0.05, n=10).

## COX5A regulates recognition via a BDNF/ERK1/2-dependent signal pathway

### *In vivo*

To determine whether COX5A could regulate BDNF to affect cognitive function, four heterozygous Tg offspring of BDNF-KD lines were established. All were able to reproduce, and were designated Founder 9, Founder 39, Founder 34, and Founder 21. Tg mice with inserted fragments, as identified by PCR, were considered BDNF-KD positive Tg (Supplementary Figure, Fig. S3-b, c).

Expressional changes of BDNF in the hippocampus of Tg with different genotypes were determined by ELISA. The results showed that BDNF protein expression was reduced by 55.23%, 41.05%, 53.55% and 1.2% in Founder 39, Founder 9, Founder 21, and Founder 34, respectively (Supplementary Figure, Fig. S3-e). The rate of protein down-regulation was calculated as follows: Rate of protein down-regulation = O.D. / O.D. WT × 100% (where the O.D. is equal to the O.D of the WT minus the O.D. of the Founder). Similar results were obtained by RT-PCR (Supplementary Figure, Fig. S3-d). Founder 39 was chosen as the target line as it showed the lowest value.

The CcO activity of hippocampal mitochondria showed that CcO activity increased in COX5A-UP Tg mice, as was described previously, and was remarkedly reduced in COX5A-UP/BDNF-KD (COX5A-UP/BDNF-KD vs WT, P=0.000007, P<0.05; COX5A-UP/BDNF-KD vs COX5A-UP, P=0.000000, P<0.05, n=10) and BDNF-KD (BDNF-KD vs WT, P=0.000015, P<0.05; BDNF-KD vs COX5A-UP, P=0.000000, P<0.05, n=10) Tg mice, as compared with that in WT and COX5A-UP lines. As shown in Figure 5, there was a significant recovery in CcO activity in COX5A-UP/BDNF-KD Tg mice when compared to that of BDNF-KD Tg mice (Supplementary Figure 3-f, COX5A-UP/BDNF-KD vs BDNF-KD, P=0.000001, P<0.05, n=10).

Measuring the ATP content exhibited a significant increase in COX5A-UP Tg mice (Supplementary Figure 3-g), and greatly decreased in COX5A-UP/BDNF-KD and BDNF-KD Tg mice when compared to that in WT and COX5A-UP lines (COX5A-UP/BDNF-KD vs WT, P=0.000013, P<0.05; BDNF-KD vs WT, P=0.000000, P<0.05; COX5A-UP/BDNF-KD vs COX5A-UP, P=0.0025, P<0.05; BDNF-KD vs COX5A-UP, P=0.00082, P<0.05, n=10). Moreover, ATP levels were restored in COX5A-UP/BDNF-KD Tg mice when compared to that in BDNF-KD Tg mice (Supplementary Figure 3-g, COX5A-UP/BDNF-KD vs BDNF-KD, P=0.00003, P<0.05, n=10).

***In vitro***

Conduction with a specific ERK1/2 inhibitor, PD98059, in COX5A-ORF or BDNF-ORF- treated neurons significantly attenuated the effects on neurite extensions induced by COX5A up-regulation (Supplementary Figure, Fig. S5). Pre-treatment with PD98059 also neutralized the dendritic restoration as induced by BDNF overexpression in the COX5A-shRNA + BDNF-ORF transfected group (BDNF rescue setting).

When compared with COX5A-ORF treatment alone, conduction with PD98059 decreased neurite extensions (P=0.00016, P<0.05, n=5) and the neuronal area (P=0.000, P<0.05, n=5) (Supplementary Figure, Fig. S5). Furthermore, transduction with PD98059 in COX5A-shRNA + BDNF-ORF neurons decreased both neurite extensions (P=0.00018, P<0.05, n=5) and the neuronal area (P=0.0008, P<0.05, n=5) when compared to COX5A-shRNA + BDNF-ORF-treated neurons. Administration of COX5A-ORF, PD98059, COX5A-shRNA, or/and BDNF-ORF had no effect on the neurite number of neurons.

# **Reference**

Kim, Y.E., Cho, H., Kim, H.J., Na, D.L., Seo, S.W., and Ki, C.S. (2020). PSEN1 variants in Korean patients with clinically suspicious early-onset familial Alzheimer's disease. *Sci Rep* 10(1)**,** 3480. doi: 10.1038/s41598-020-59829-z.

Ratliff, W.A., Mervis, R.F., Citron, B.A., Schwartz, B., Rubovitch, V., Schreiber, S., et al. (2020). Effect of mild blast-induced TBI on dendritic architecture of the cortex and hippocampus in the mouse. *Sci Rep* 10(1)**,** 2206. doi: 10.1038/s41598-020-59252-4.

# Supplementary Figures and Tables

## Supplementary Figures

**
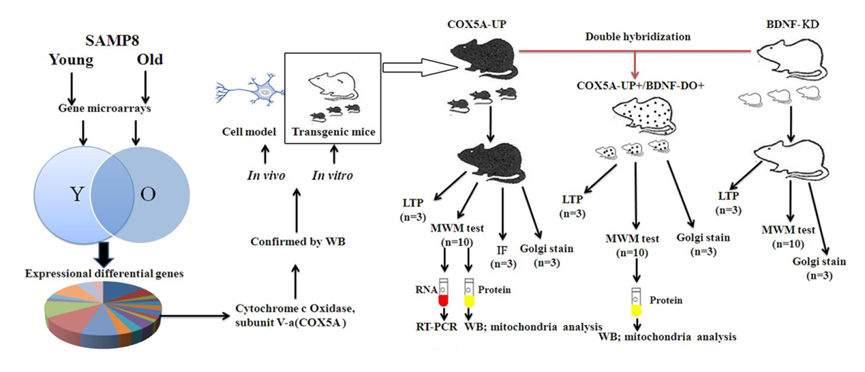
**

**Supplementary Figure S1: Animal grouping and laboratory investigations**

Shown are the animal grouping and laboratory investigations in the present study. Abbreviations: SAMP8, senescence-accelerated mouse-prone 8, Y, young; O, old; WB, Western blot analysis; LTP, long-term potentiation; RT-PCR, reverse transcription-polymerase chain reaction; MWT, Morris Water Test; IF, immunofluorescence.


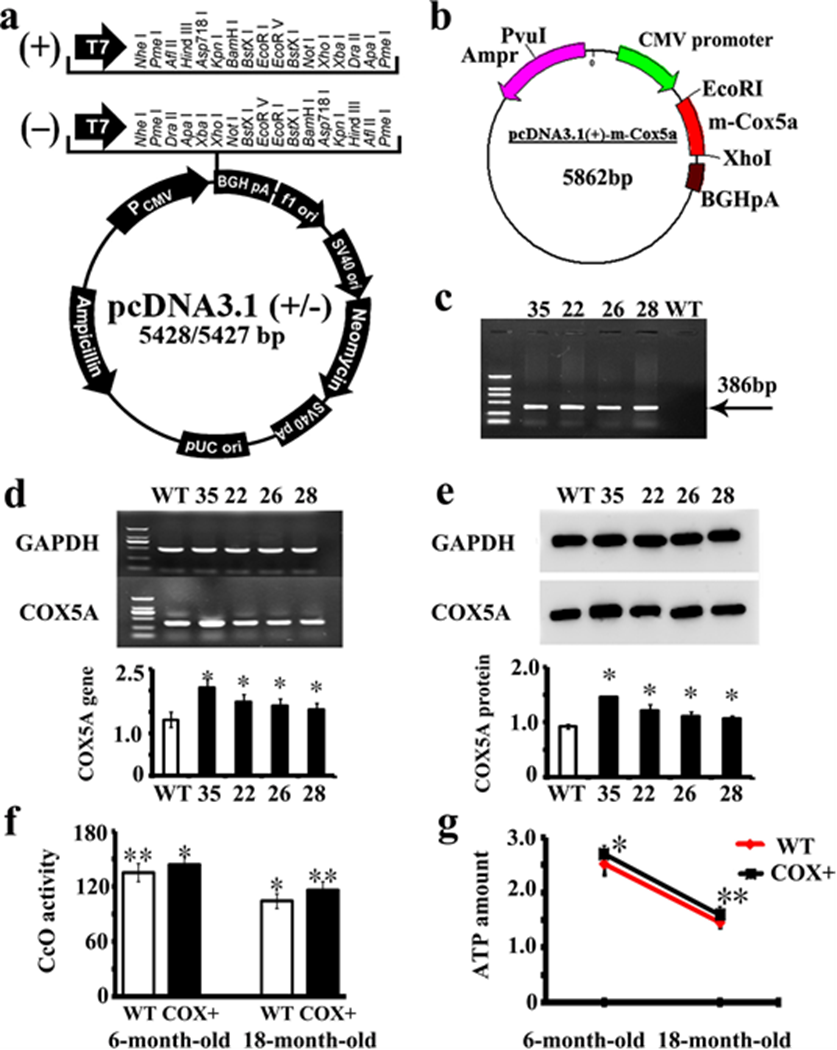


**Supplementary Figure S2: Newly development Tg mice with COX5A overexpression.**

**(a)** and **(b)**, recombination plasmid for COX5A up-regulation. **(c)**, Lane 1: DNA marker DL 2,000. Lane 2 to lane 6: Founder 35, 22, 26, 28, and WT, respectively. **(d)**, mRNA expression of COX5A in the mouse hippocampus. **(e)**, protein expression and quantity analysis of COX5A detected by WB in the hippocampus. * vs WT mice, P<0.05. **(f)**, CcO activity in hippocampal mitochondria from both WT and COX5A-UP Tg mice at 6 and 18 months. **(g)**, the ATP content measured in mitochondria. * vs 6m-old WT mice, P<0.05; ** vs 18m-old WT mice, P<0.05. WT, wild type mice; COX+, COX5A overexpressing Tg mice.


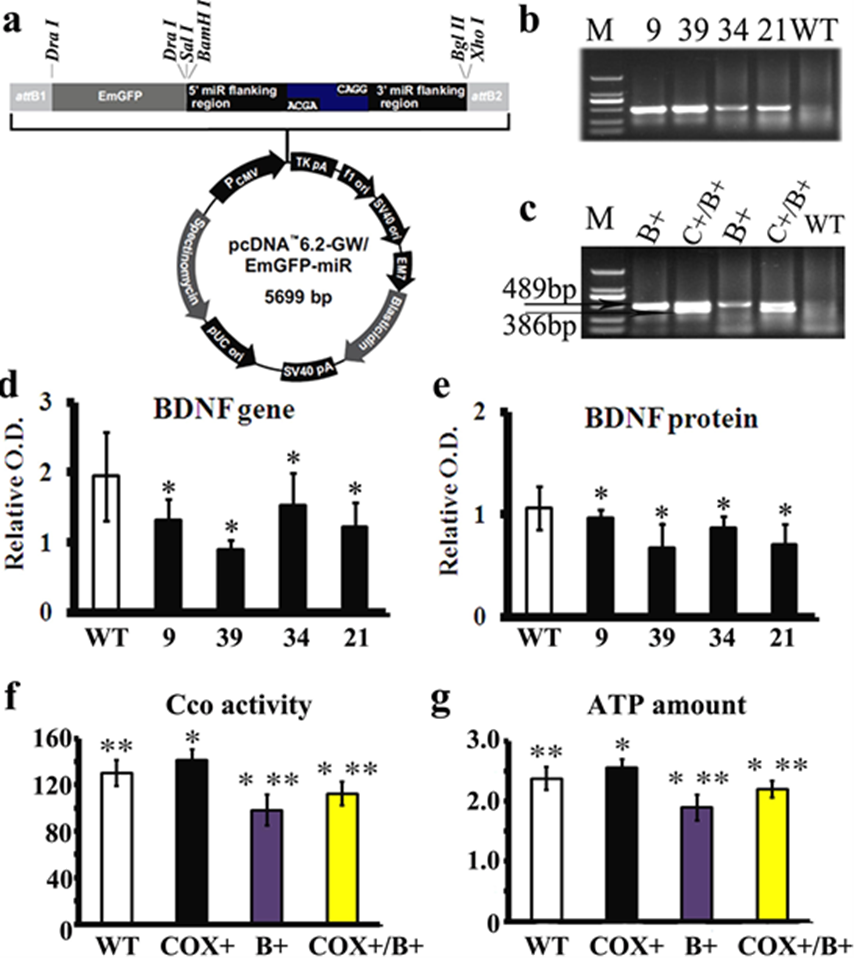


**Supplementary Figure S3: Newly established transgenic mice that overexpress COX5A and downregulate BDNF**

WT, wild type mice; COX+, COX5A-UP transgenic (Tg) mice; B+, BDNF-KD Tg mice;

COX+/B+, COX5A-UP/BDNF-KD Tg mice

**(a)**, the illustration of pcDNA3.1 (+) for BDNF knockdown. **(b)**, M: DNA marker DL 2,000. Lane 2-6: Founder 9, 39, 34, 21, and WT, respectively. **(c)**, M: DNA marker DL 2,000. Lane 2-5: Founder 53, 67, 19, 4, and WT, respectively. The mRNA **(d)** and protein **(e)** expression of BDNF detected in the hippocampus of BDNF-KD Tg and WT mice. **(f)**, the CcO activity in hippocampal mitochondria of BDNF-KD, COX5A-UP/BDNF-KD Tg, and WT mice. **(g)**, the ATP content was evaluated in mitochondria from the mice described above. Values plotted indicate the mean ± SD (n=10).

* vs WT mice, P<0.05; ** vs COX5A-UP Tg mice.


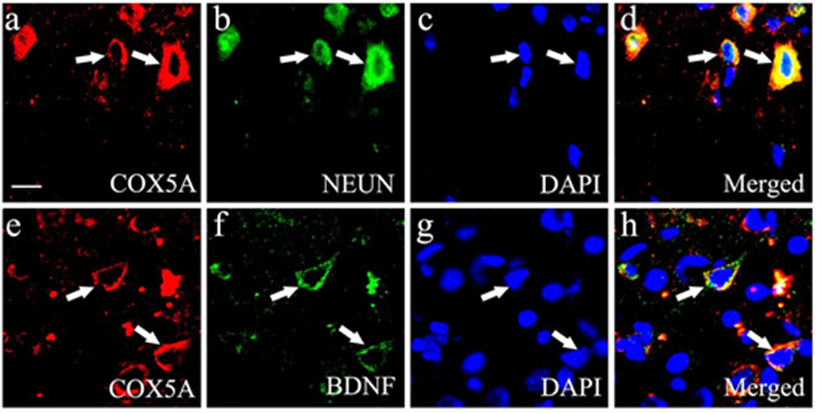


**Supplementary Figure S4 Expression and localization of COX5A and BDNF in the mouse hippocampus**

**(a-d)**, hippocampal COX5A co-localization with neurons **(a-d)** and BDNF **(e-h)**. **(a)** and **(e)**, COX5A staining (red); **(b)**, immunofluorescence (IF) of NeuN staining (green); **(c)** and **(g)**, DAPI staining (blue); **(f)**, BDNF staining (green); **(d)** and **(h)**, merged image.

Magnifications, 400×; Scale bar, 5 µm.


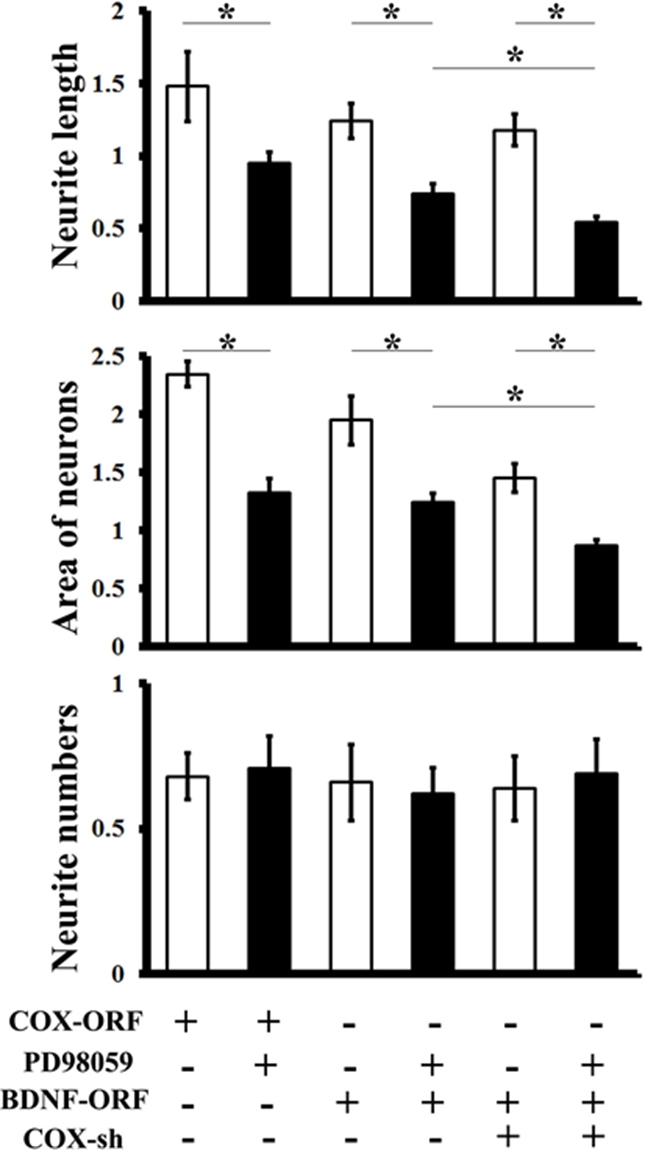


**Supplementary Figure S5: Effects of COX5A via the BDNF/ERK1/2-dependent pathway *in vitro***

Quantification of neurite length, the areas of cultured neurons, and the number of neurites after introduction with COX5A-ORF, ERK1/2 inhibitor PD98059, COX5A-shRNA, BDNF-ORF, and/or COX5A-shRNA, respectively. Values plotted indicate the mean ± SD. *, P<0.05.

COX-ORF, COX5A-ORF; COX-sh, COX5A-shRNA.


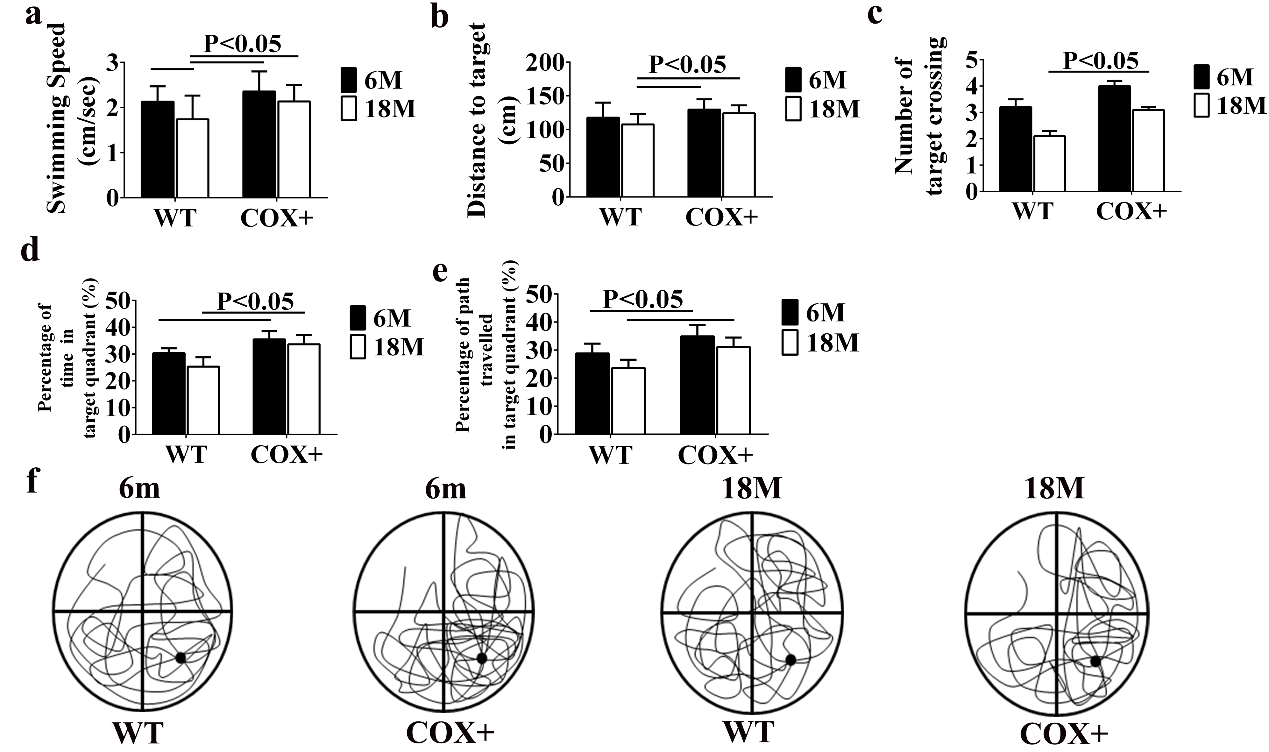


Supplementary Figure S6. Effects of COX5A on the spatial learning and memory changes during probe trail of Morris water maze. (a) Average swimming speed, (b) distance, (c) number of target crossing, (d) percentage of time target quadrant, (e) percentage of path travelled in target quadrant and (f) typical probe traces evaluated by the Morris Water Maze (MWM) test in the 6-months-old (6M) and 18-months-old (18M) COX5A-UP transgenic (Tg) and the age-matched WT mice.

## Supplementary Tables

**Supplementary Table S1: Cell grouping and treatment. Cultured neurons were prepared and treated as described in** **the Table.**

|  | **PB** | **Vector-ORF** | **COX5A-ORF** | **BDNF-ORF** | **Vector-shRNA** | **COX5A-shRNA** | **BDNF-shRNA** |
| --- | --- | --- | --- | --- | --- | --- | --- |
| **Blank** | 一 | 一 | 一 | 一 | 一 | 一 | 一 |
| **Control-PB** | 十 | 一 | 一 | 一 | 一 | 一 | 一 |
| **Control-ORF** | 一 | 十 | 一 | 一 | 一 | 一 | 一 |
| **COX5A**  **overexpression** | 一 | 一 | 十 | 一 | 一 | 一 | 一 |
| **BDNF overexpression** | 一 | 一 | 一 | 十 | 一 | 一 | 一 |
| **Control-shRNA** | 一 | 一 | 一 | 一 | 十 | 一 | 一 |
| **COX5A**  **down-regulation** | 一 | 一 | 一 | 一 | 一 | 十 | 一 |
| **BDNF rescue 1** | 一 | 一 | 一 | 十 | 一 | 十 | 一 |
| **BDNF rescue 2** | 一 | 一 | 十 | 一 | 一 | 一 | 十 |
| **BDNF rescue 3** | 一 | 一 | 十 | 十 | 一 | 一 | 十 |

Normal, blank; PB, phosphate buffer; Control-ORF, Vector-ORF; COX5A-ORF, COX5A overexpression; BDNF-ORF, BDNF overexpression; Control-shRNA, Vector-shRNA; COX5A-shRNA, COX5A down-regulation; BDNF-shRNA, BDNF down-regulation.

**Supplementary Table S2-1 Effects of COX5A overexpression on spatial learning evaluated by MWM test in mice**

**(mean ± SD, n=10)**

| **Escape latency (s)** | | **Days post hiding platform (days)** | | | | | | |
| --- | --- | --- | --- | --- | --- | --- | --- | --- |
|  |  | **1d** | **2d** | **3d** | **4d** | **5d** | **F values** | **P values** |
| **6m-old** | **WT** | 43.21±7.14 | 40.63±7.27 | 34.27±6.31 | 31.18±5.22 | 28.54±5.47 | 16.84 | 0.001 |
|  |  | | | | | | | |
|  | **COX+** | 39.11±6.32 | 32.87±5.98 | 27.47±4.13 | 24.36±6.27 | 21.34±4.18 | 30.15 | 0.000 |
|  |  | | | | | | sum 28.14 | 0.000 |
|  | **t values** | 0.125 | 8.624 | 7.005 | 8.527 | 9.548 |  |  |
|  | **P values** | 0.927 | 0.000 | 0.000 | 0.000 | 0.000 |  |  |
| **18m-old** | **WT** | 55.06±6.67 | 51.52±8.26 | 42.73±9.17 | 38.26±4.62 | 33.52±5.02 | 18.64 | 0.001 |
|  |  |  |  |  |  |  |  |  |
|  | **COX+** | 49.58±7.24 | 40.91±6.25 | 34.68±5.68 | 28.73±5.88 | 24.65±5.17 | 39.51 | 0.000 |
|  |  |  |  |  |  |  | sum 30.17 | 0.000 |
|  | **t values** | 0.225 | 9.578 | 8.224 | 9.045 | 7.589 |  |  |
|  | **P values** | 0.847 | 0.000 | 0.000 | 0.000 | 0.000 |  |  |

**Supplementary Table S2-2 Effects of age on spatial learning evaluated by MWM test in WT and Tg mice**

**(mean ± SD, n=10)**

| **Escape latency (s)** | | **Days post hiding platform (days)** | | | | | | |
| --- | --- | --- | --- | --- | --- | --- | --- | --- |
|  |  | **1d** | **2d** | **3d** | **4d** | **5d** | **F values** | **P values** |
| **WT** | **6m-old** | 43.21±7.14 | 40.63±7.27 | 34.27±6.31 | 31.18±5.22 | 28.54±5.47 | 16.84 | 0.001 |
|  |  | | | | | | | |
|  | **18m-old** | 55.06±6.67 | 51.52±8.26 | 42.73±9.17 | 38.26±4.62 | 33.52±5.02 | 18.64 | 0.001 |
|  |  | | | | | | sum 12.58 | 0.001 |
|  | **t values** | 7.125 | 9.336 | 8.258 | 7.154 | 6.558 |  |  |
|  | **P values** | 0.000 | 0.000 | 0.000 | 0.000 | 0.000 |  |  |
| **COX+** | **6m-old** | 39.11±6.32 | 32.87±5.98 | 27.47±4.13 | 24.36±6.27 | 21.34±4.18 | 30.15 | 0.000 |
|  |  |  |  |  |  |  |  |  |
|  | **18m-old** | 55.06±6.67 | 51.52±8.26 | 42.73±9.17 | 38.26±4.62 | 33.52±5.02 | 39.51 | 0.000 |
|  |  |  |  |  |  |  | sum 33.58 | 0.000 |
|  | **t values** | 5.224 | 8.247 | 10.334 | 9.665 | 7.214 |  |  |
|  | **P values** | 0.000 | 0.000 | 0.000 | 0.000 | 0.000 |  |  |

**Supplementary Table S3 COX5A up-regulation rescues hippocampal synaptic LTP decline with age**

| **fEPSPslope (%)** | **Age** | | | | | |
| --- | --- | --- | --- | --- | --- | --- |
| **Genotype** | **6-m old** | **18-m old** | | **F values** | | **Pvalues** |
| **WT** | 147.52±20.11 | 123.56±15.65 | 19.25 | | | 0.000 |
| **COX+** | 188.14±19.27 | 142.38±25.29 | 31.28 | | | 0.000 |
|  |  |  | Sum 21.36 | | | 0.000 |
| **F values** | 30.25 | 14.21 | Sum 25.24 | | |  |
| **P values** | 0.000 | 0.000 |  | | 0.000 |  |

**Supplementary Table S4 Effects of COX5A on spatial learning by BDNF evaluated by MWM test**

**(mean ± SD, n=10)**

| **Escape latency (s)** | **Days post hiding platform (days)** | | | | | | |
| --- | --- | --- | --- | --- | --- | --- | --- |
|  | 1d | 2d | 3d | 4d | 5d | **F values** | **P values** |
| **WT** | 39.87±6.39 | 36.32±5.24 | 30.55±2.67 | 28.7±2.12 | 27.32±4.26 | **18.84** | **0.000** |
| **COX+** | 39.11±2.31 | 32.87±3.02 | 27.47±1.34 | 24.36±2.12 | 21.34±3.28 | **25.12** | **0.000** |
| **B+** | 46.62±4.36 | 41.73±1.35 | 37.27±2.38 | 34.65±2.29 | 31.68±4.87 | **20.64** | **0.000** |
| **COX+/B+** | 41.31±4.32 | 37.45±5.98 | 34.63±4.13 | 29.46±2.27 | 28.71±4.18 | **19.32** | **0.000** |
| **t values (v) B+ vs WT** | 8.212 | 8.367 | 9.327 | 6.328 | 5.124 | **sum 21.36** | **0.000** |
| ***P* values (v)** | 0.000 | 0.000 | 0.000 | 0.000 | 0.000 |  |  |
| **tv B+ vs COX+** | 7.655 | 9.658 | 10.254 | 9.351 | 9.117 |  |  |
| ***P*v** | 0.000 | 0.000 | 0.000 | 0.000 | 0.000 |  |  |
| **tv B+vsCOX+/B+** | 6.325 | 8.021 | 7.328 | 6.214 | 5.368 |  |  |
| ***P*v** | 0.000 | 0.000 | 0.000 | 0.000 | 0.000 |  |  |
| **tv COX+vsCOX+/B+** | 0.212 | 5.324 | 7.254 | 4.068 | 6.247 |  |  |
| ***P*v** | 0.08 | 0.000 | 0.000 | 0.000 | 0.000 |  |  |
| **tv COX+/B+ vs WT** | 0.012 | 0.324 | 2.147 | 0.068 | 0.047 |  |  |
| ***P*v** | 0.76 | 0.85 | 0.011 | 0.84 | 0.92 |  |  |

**Supplementary Table S5 COX5A up-regulation rescues hippocampal synaptic LTP decline in BDNF knockdown mice**

| **Genotype** | **fEPSPslope (%)** |
| --- | --- |
| **WT** | 131.37± 8.03 |
| **COX+** | 156.92± 5.33 |
| **BDNF+** | 106.03± 3.99 |
| **BDNF+/COX+** | 125.81± 8.64 |
| **F values** | 20.36 |
| **Pvalues** | 0.000 |

**Table S6 Protein expressional changes with age in WT and Tg mice**

**(mean ± SD, n=10)**

* vs WT 6-m, P<0.05; # vs COX+ 18-m, P<0.05; & vs COX+ 6-m, P<0.05

| **Protein** | **WT** | | **COX+** | |  |
| --- | --- | --- | --- | --- | --- |
|  | **6-m** | **18-m** | **6-m** | **18-m** |  |
| **COX5A** | 0.68±0.09 | 0.32±0.02*# | 1.26±0.07* | 1.02±0.11& |  |
| **BDNF** | 0.42±0.02 | 0.23±0.05*# | 0.61±0.05* | 0.48±0.02& |  |
| **ERK1/2** | 0.86±0.03 | 0.70±0.02*# | 1.06±0.11* | 0.91±0.04& |  |
| **p-ERK1/2** | 0.21±0.01 | 0.14±0.01*# | 0.62±0.01* | 0.44±0.02& |  |
